# A novel electrochemical nanobiosensor for the ultrasensitive and specific detection of femtomolar-level gastric cancer biomarker miRNA-106a

**DOI:** 10.3762/bjnano.7.193

**Published:** 2016-12-19

**Authors:** Maryam Daneshpour, Kobra Omidfar, Hossein Ghanbarian

**Affiliations:** 1 Biotechnology Department, School of Medicine, Shahid Beheshti University of Medical Sciences, Tehran, Iran; 2 Biosensor Research Center, Endocrinology and Metabolism Molecular-Cellular Sciences Institute, Tehran University of Medical Sciences, Tehran, Iran,; 3 Endocrinology and Metabolism Research Center, Endocrinology and Metabolism Research Institute, Tehran University of Medical Sciences, Tehran, Iran; 4 Cellular and Molecular Biology Research Center, Shahid Beheshti University of Medical Sciences, Tehran, Iran; 5 Department of Biotechnology, School of Advanced Technologies in Medicine, Shahid Beheshti University of Medical Sciences, Tehran, Iran

**Keywords:** electrochemical nanobiosensor, gastric cancer, gold–magnetic nanoparticle, miR-106a

## Abstract

Gastric cancer (GC) is the second leading cause of cancer-related deaths all over the world. miR-106a is a circulatory oncogenic microRNA (miRNA), which overexpresses in various malignancies, especially in GC. In this study, an ultrasensitive electrochemical nanobiosensor was developed for the detection of miR-106a using a double-specific probe methodology and a gold–magnetic nanocomposite as tracing tag. The successful modification of the electrode and hybridization with the target miRNA were confirmed by electrochemical impedance spectroscopy (EIS) and cyclic voltammetry (CV) methods. Differential pulse voltammetry (DPV) was used for quantitative evaluation of miR-106a via recording the reduction peak current of gold nanoparticles. The electrochemical signal had a linear relationship with the concentration of the target miRNA ranging from 1 × 10^−3^ pM to 1 × 10^3^ pM, and the detection limit was 3 × 10^−4^ pM. The proposed miRNA-nanobiosensor showed remarkable selectivity, high specificity, agreeable storage stability, and great performance in real sample investigation with no pretreatment or amplification. Consequently, our biosensing strategy offers such a promising application to be used for clinical early detection of GC and additionally the screen of any miRNA sequence.

## Introduction

Gastric cancer (GC) is one of the most lethal malignancies and the second leading cause of cancer mortality worldwide [1]. In spite of recent advances in diagnostic techniques and pre-operative management, the prognosis of patients with advanced disease is still poor, even in the developed countries [2]. The most commonly used tumor markers such as carcinoembryonic antigen (CEA) and cancer antigen 19-9 (CA19-9) have insufficient sensitivity and specificity, thus limiting their application in early diagnosis of GC [3]. Therefore, the establishment of novel strong specific biomarkers with sufficient sensitivity is an ideal strategy for improving the early detection of GC and the cure rates for patients. Besides, these biomarkers should be easy to evaluate and reliably associated with clinical outcomes. Discovery of miRNAs and the approval of their role in tumorigenesis along with the development of various cancers have presented them as the suitable cancer biomarkers [4]. There is also growing evidence that miRNAs exist not only in cells but also in a variety of body fluids, including blood [5], saliva [6], and urine [7]. Those miRNAs that can be found in bloodstream are called circulatory miRNAs. They are generally cancer-specific and their expression patterns are greatly comparable between patient and healthy persons. Moreover, it has been demonstrated that the circulatory miRNAs are noticeably resistant to RNase digestion, non-physiologic pH values, and high temperature. Hence, these miRNAs have been considered as promising biomarker for early cancer detection [8–9].

There are already numerous reports that specify various miRNAs involved in GC tumorigenesis such as miR-106a [3]. miR-106a belongs to the miR-17 family and is an oncogenic member of miR-106a-363 cluster which is located on Xq26.2. Unlike most members of this cluster, the altered expression of miR-106a can be followed in solid tumors, stool, and plasma/serum samples of patients with gastrointestinal tumors such as GC and colorectal cancer [10–11]. According to the recent studies, miR-106a level is significantly associated with tumor node metastasis (TNM) stage, tumor size and differentiation, lymphatic and distant metastasis, and invasion [10–12].

The clinical importance of miRNAs has consequently fueled the urgent demand for developing reliable and ultrasensitive diagnostic techniques to detect these valuable biomarkers for early cancer diagnosis and prognosis. So far, different strategies have been developed in this regard, such as quantitative reverse transcription PCR (qRT-PCR) [13], locked nucleic acid-based northern blot [14], microarray [15], or flowcytometry [16]. Although there have been advantages to each of these methods, none of them meets the high standards for clinical evaluation of miRNAs. Furthermore, almost all these techniques suffer from some known disadvantages including long analysis time, expensive reagents, high requirements to technical equipment, lack of adequate specificity, and poor reproducibility [9]. On the other hand, the technical challenges for the quantitative analysis of miRNA become more complex due to their small size, low abundance, and high sequence similarity. In an effort to resolve the mentioned limitations, biosensor technology was suggested as an alternative method for sensitive, specific and non-invasive miRNA detections in early cancer diagnosis [8]. Biosensors are commonly defined as devices composed of recognition elements interfaced with a transducer, which finally transform a capture process into a detectable output signal. This signal, which is almost always the result of a specific bio-interaction, could be received as an evident change in color, pH value, or electrochemical properties of the microenvironment [17–18].

Among all types of biosensors, electrochemical sensors have been of great interest particularly because they are simple, portable, sensitive, inexpensive, and offer a fast response [19]. Using electrochemical methodologies, the change in the amount of transferred ions in the presence of a target molecule can simply be related to its concentration. The highly flexible nature of these devices allows for applying various modifications to provide ultrasensitive quantitative measurements of specific biomolecules and even simultaneous evaluation of multiple analytes [19]. In addition, some novel approaches including the use of nanomaterials [20–22], semi-conductive/conductive polymers [23], and enzymatic strategies [24–26] have been recently employed to improve the detection sensitivity and also the stability of the electrochemical biosensors. It has been shown that these approaches can strongly amplify the signal, extend the linear detection range, and lower the detection limit of the assay [19]. Nowadays, electrochemical nanobiosensors are an emerging field of biosensors that unites the unique properties of nanomaterials and the advantages of electrochemical sensing to raise the efficacy, sensitivity, stability, and reproducibility of the analytical miniature devices [27].

Over the past years, miRNAs have been one of the most attractive targets for the construction of electrochemical nanobiosensors [8,21,28]. There are numerous studies on fabricating electrochemical nanobiosensors for the detection of label-free or labeled miRNA. Although biosensors based on electrochemical impedance spectroscopy (EIS) offered a label-free methodology and reached a low detection limit for miRNA [29], most of the label-free miRNA biosensors are developed using molecular probes such as methylene blue [30], Oracet blue [21], and ferrocene boronic acid [31].

In spite of the quicker and more accurate results offered by the label-free methods, it seems that labeled approaches provide more feasible options for overcoming the difficulty of detecting small miRNAs [19]. Based on this idea, various miRNA-biosensors have been fabricated using different enzymes [32], nanomaterials [33], and enzyme–nanomaterial combinations [34]. Due to unique electrochemical characteristics, high surface-to-volume ratio, remarkable surface energy, and great biocompatibility, gold nanoparticles have been one of the most common choices for labeling in electrochemical biosensors [35].

In the present study, we fabricated a simple, sensitive, and specific electrochemical nanobiosensor for the detection of miR-106a using gold–magnetic NPs. Gold–magnetic NPs were primarily decorated with single-strand (ss)-probe 1 (P1) to form a nanoprobe. The nanoprobe was then used for separating the miRNA target from the sample solution via the magnetic properties of the Fe_3_O_4_ NPs. At the last step of assay, the nanoprobe–miRNA hybrid was captured by the ss-probe 2 (P2), which was previously immobilized on the surface of a screen-printed carbon electrode (SPCE). While the miR-106a target is hybridized with both probes in a sandwich format, the gold–magnetic NPs would generate the final signal due to the presence of gold NPs.

To the best of our knowledge, there is no report on using this kind of nanocomposite for the fabrication of a miRNA biosensor. In addition, we could not find any research on biosensor development for the detection miR-106a or other GC-specific biomarkers. Hence, the simple and sensitive miR-106a nanobiosensor proposed here may be well suited for the early detection of GC.

## Results and Discussion

In this study, a simple efficient nanobiosensor was developed to detect trace amounts of miR-106a as a reliable biomarker for GC. With the combination of a well-characterized nanomaterial and the complementarity principle of nucleic acid molecules, this electrochemical nanobiosensor showed remarkable accuracy in evaluating miRNA target concentrations via differential pulse voltammetry (DPV) analyses.

**Figure 1:**
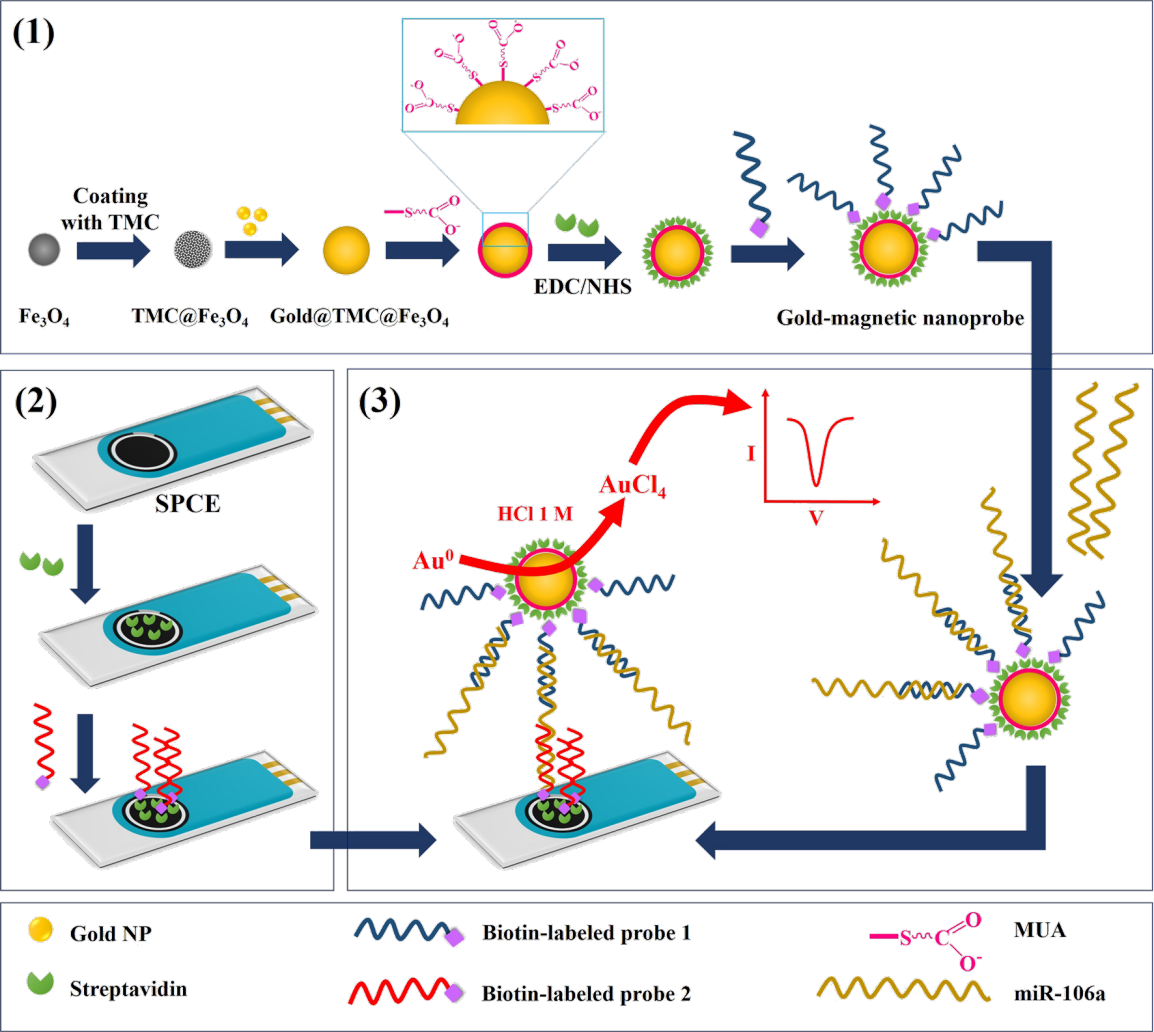
Schematic of the principal mechanism for miR-106a detection by the nanobiosensor. (1) Preparation the nanoprobe; (2) modification of the electrode; and (3) hybridization steps (TMC = *N*-trimethylchitosan).

The principal steps of the biosensing procedure are schematically shown in Figure 1. After synthesis of gold–magnetic NPs as a three-layer nanocomposite, these particles were covalently coated with streptavidin by using 11-mercaptoundecanoic acid (MUA) and carbodiimide hydrochloride/*N*-hydroxysulfosuccinimide (EDC/NHS). Next, P1 was immobilized on NPs through streptavidin–biotin interaction. In parallel, the working area of SPCE was covered by streptavidin and then exposed to P2. In order to quantify miR-106a in samples, the target was first captured by gold–magnetic nanoprobes and then hybridized with P2 immobilized on the electrode. The final detection was carried out by DPV measurements based on redox reactions of the gold NPs in the nanoprobe/target/P2 complex.

### Characterization of gold–magnetic NPs

**Figure 2:**
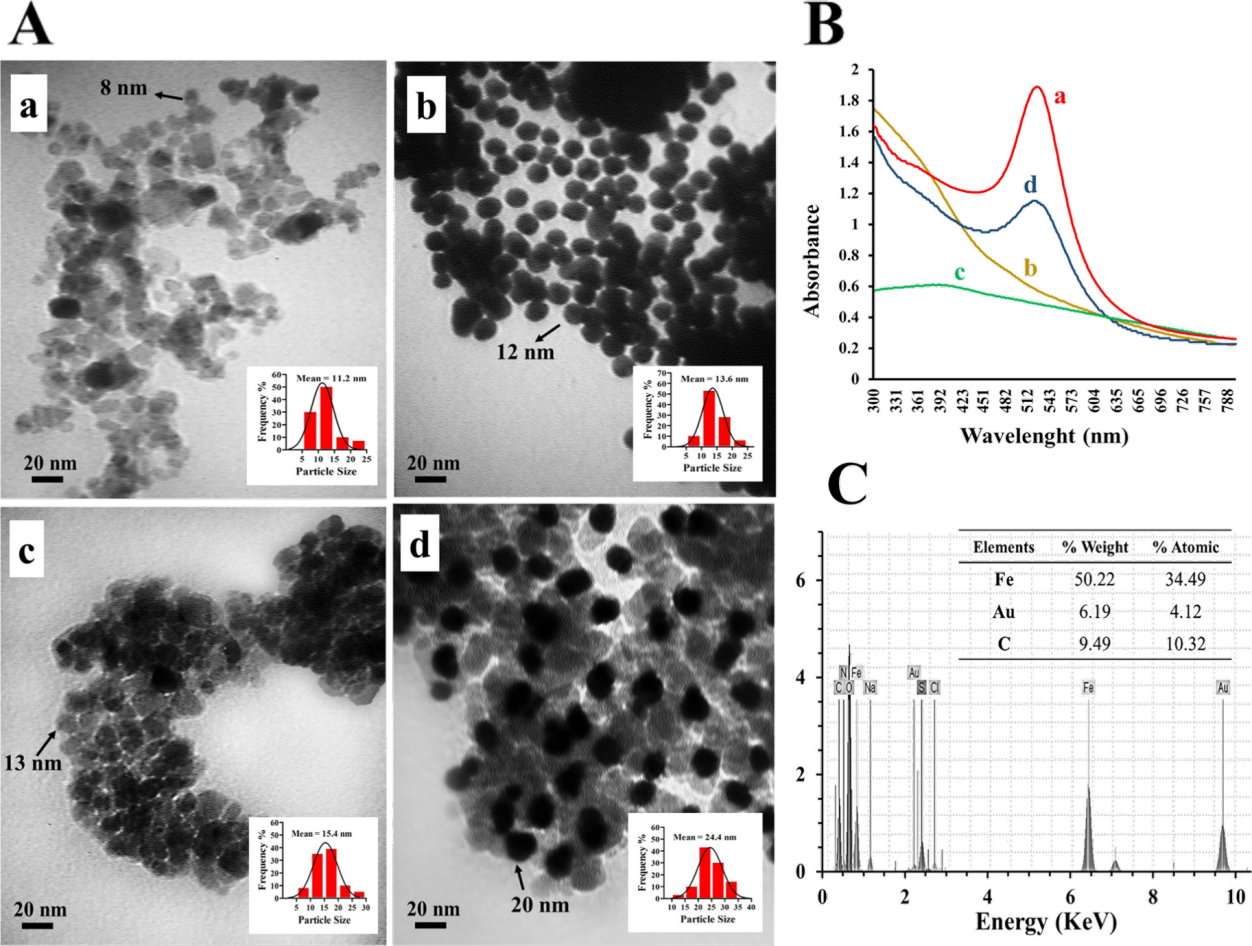
(A) TEM images of synthesized Fe_3_O_4_ NPs (a), gold NPs (b), TMC@Fe_3_O_4_ NPs (c), and gold–magnetic NPs (d) with their corresponding particle size distribution (inset). (B) UV–vis analysis of gold NPs (a), Fe_3_O_4_ NPs (b), TMC@Fe_3_O_4_ NPs (c), and gold–magnetic NPs (d). (C) EDXD spectra of gold–magnetic NPs.

The size and morphology of all the produced NPs were investigated using TEM. The synthesized uncoated Fe_3_O_4_ NPs with an average diameter of 10 nm can be observed in Figure 2A(a). These NPs were essentially monodisperse and partially aggregated because of their magnetic nature. Figure 2A(b) shows the gold NPs with a mean diameter of 12 nm and spherical morphology. TEM results of the magnetic TMC@Fe_3_O_4_ NPs demonstrated their homogenous structure with a mean diameter of 13 nm (Figure 2A(c)). Presumably, the Fe_3_O_4_ cores were well covered by an intermediate coating layer of *N*-trimethylchitosan (TMC) with a thickness of about 2–3 nm during the synthesis process. The importance of this step is that using TMC for covering the magnetic particles does not increase massively the particle size, which is a common undesirable result from using most other polymers [36–38]. In Figure 2A(d), the presence of gold NPs is obvious through the high contrast against other particles and background. The mean diameter of the final nanocomposites has reasonably increased after the assembly of gold NPs and reached 20 nm. The estimated sizes from TEM images showed a good agreement with the DLS measurements (the insets).

The UV–vis absorption spectrum of a colloidal solution of gold NPs is presented in Figure 2B and compared with the ones resulted from other synthesized NPs. The results indicated a remarkable peak near 520 nm in the gold (curve a) and final gold–magnetic (curve d) NPs analysis. This peak, which forms because of the excitation of surface plasmon vibrations and corroborates the presence of spherical gold NPs, is predictably absent in UV–vis absorption spectrum of Fe_3_O_4_ and TMC@Fe_3_O_4_ NPs.

Furthermore, the chemical composition of the particles was studied via EDX analysis. The EDX spectrum (Figure 2C) verified the presence of Fe, O, and Au. The considerable amounts of H, C, and N in the nanocomplex represent the TMC polymer and indicate the successful formation of desired gold@TMC@Fe_3_O_4_ NPs.

In order to verify the successful assembly of TMC-coated Fe_3_O_4_ NPs and final gold–magnetic nanocomposites, the TMC polymer, Fe_3_O_4_, TMC@Fe_3_O_4_, and gold@TMC@Fe_3_O_4_ NPs were subjected to FTIR analysis. As Figure S1 (Supporting Information File 1) shows, the FTIR spectrum of TMC (curve a) indicates four typical peaks at about 3430 cm^−1^ (overlap of O–H and intermolecular hydrogen bonding vibration), 2900 cm^−1^ (C–H bond), 1650 cm^−1^ (N–H bending vibration), and 1070 cm^−1^ (C–O–C stretching vibration). In addition, there are two specific peaks around 1470 and 1600 cm^−1^, which can be considered for differentiating TMC from chitosan. The second spectrum is obtained from naked Fe_3_O_4_ NPs and included a strong peak at about 585 cm^−1^, which correlates to Fe–O. This peak can be also observed in FTIR spectra obtained from TMC@Fe_3_O_4_, and gold@TMC@Fe_3_O_4_ NPs (curve c and d, respectively). Furthermore, the shifted N–H bond has formed a detectable peak at 1502 cm^−1^ in the spectrum of TMC@Fe_3_O_4_ NPs. This curve contains another notable peak at about 1630 cm^−1^ which arises from the interaction between the N–H groups of TMC and the CHO group of glutaraldehyde known as “Schiff base formation”. As it can be seen, there is no additional specific peak in the last spectrum (curve d) and the assembly of gold NPs on the surface of TMC@Fe_3_O_4_ NPs has just attenuated the existing peaks [36,39].

According to the Figure S2A (Supporting Information File 1), the XRD pattern of synthesized gold–magnetic particles confirms not only the crystallinity of the nanostructures but also the presence of gold in the synthesized nanocomposite. The XRD result shows six significant reflections of the (220), (311), (422), (511), (440), and (533) plane spacings of the magnetite phase with spinel structure (JCPDS card no. 79-0418). The additional peaks appeared at (111), (200), (220) and (311) are related to gold NPs assembled onto the magnetic core (JCPDS card no. 04-0784) [40].

Since the gold–magnetic NPs primarily act as the separator, their magnetic behavior influences their efficacy and performance in the assay. Hence, the magnetization curve of prepared NPs was studied by Vibrating sample magnetometry (VSM) at room temperature. The hysteresis curves in Figure S2B (Supporting Information File 1) show that the saturation magnetization of naked magnetic NPs (70.62 emu/g) has decreased after coating with TMC polymer and gold NPs. However, the zero remanence and coercivity values indicated the superparamagnetic nature of gold–magnetic NPs with an acceptable VSM value (59.05 emu/g) [20].

### Preparation of gold–magnetic nanoprobes

The oligonucleotides can be immobilized on the gold NPs through various strategies such as using the thiol group, particle functionalization, and streptavidin–biotin interaction. Streptavidin has an extraordinary high affinity for biotin and the streptavidin–biotin bond is one of the strongest non-covalent interactions known in nature (with a dissociation constant in the order of 4 × 10^−14^ M) [41]. Therefore, we used this strategy in preparing gold–magnetic nanoprobes. Immobilization of P1 on the gold–magnetic NPs was initiated by functionalizing these particles using MUA prior the streptavidin coating in presence of EDC/NHS. Each molecule of MUA provides one biding site through its carboxylic group, which is then activated by EDC in combination with NHS [42]. The OD_280_ values of pre- and post-coupling showed the efficacy of 88% for streptavidin binding to the functionalized NPs. Compared to our previous work [43], in which the passive adsorption via electrostatic and hydrophobic interaction between streptavidin and gold-magnetic NPs was used, the covalent binding methods provided more stable chemical bonds and higher binding efficacy. The main parameters that should be carefully controlled when using EDC/NHS are the pH value, the EDC/NHS ratio, and the amount of EDC that prevents the NPs aggregation due to loss of electrostatic repulsive forces [44]. As it is proposed in the product data sheet, we adjusted the pH value at about 6 and used an EDC/NHS molar ratio of 1:2.

Subsequently, streptavidin-coated NPs were incubated with biotin-labeled P1. The interaction between streptavidin and biotin is remarkably fast and stable and leads to non-covalent immobilization of P1 onto the NPs. Finally, an immobilization efficacy of about 90% was achieved based on the measured OD_260_ values before and after immobilization. Improved immobilization efficacy compared to our previous work revealed that better streptavidin coating at the primer step can significantly result in higher probe immobilization efficacy and surely elevate the sensitivity and stability of following biosensing procedure.

### Characterization of modified electrodes and assessment of the fabrication procedure

**Figure 3:**
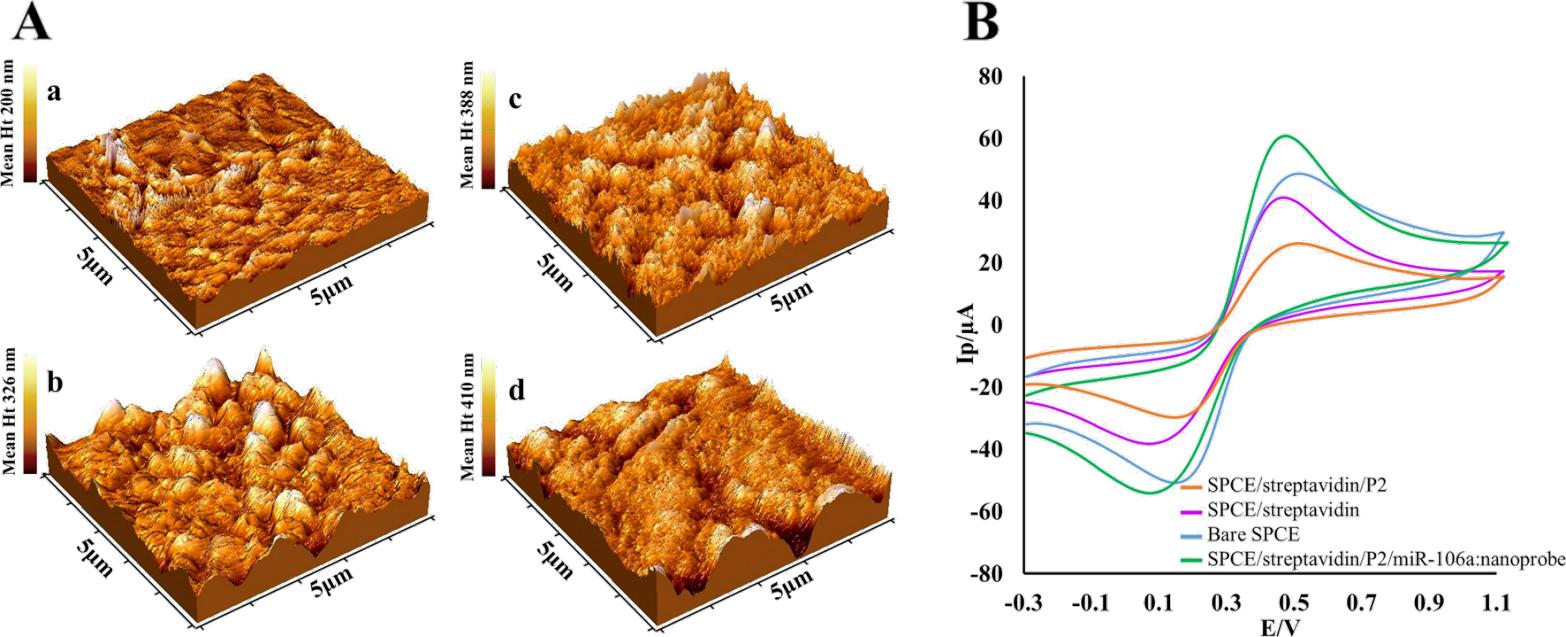
(A) AFM images of (a) a bare SPCE, (b) a SPCE after coating with streptavidin, (c) after immobilization of P2, and (d) after hybridization with the target complex. (B) Cyclic voltammograms carried out in 5.0 mM [Fe(CN)_6_]^3−/4−^ solution containing 1.0 M KCl, at a scan rate of 100 mV/s for bare SPCE (blue), streptavidin-coated SPCE (purple), P2/streptavidin-coated SPCE (orange), and after hybridization with the target complex (green).

The streptavidin–biotin approach was also used to immobilize P2 on the electrode surface. For this purpose, the SPCEs were modified by physical attachment of streptavidin and immobilization of biotinylated P2. Topology alterations occurred on the SPCE surface during mentioned modifications were explored using AFM. Figure 3A shows the AFM pictures obtained for a bare SPCE, as well as for an SPCE after coating with streptavidin, after immobilization of P2, and after the hybridization of the target complex (gold–magnetic nanoprobe/miR-106a), respectively. The mean height of the deposits clearly shows that the thickness of assembled layers increased step by step. Moreover, the roughness of SPCE surface notably changed after coating with streptavidin and a nodular texture appeared. Further immobilization with P2 introduced a needle-like structure to the surface and enhanced the average roughness. The surface of the SPCE reformed after the hybridization of the target complex resulting in the smoother topography of the surface.

Furthermore, to ensure the accuracy of the nanobiosensor preparation procedure, CV measurements were carried out after each assembly step. Figure 3B illustrated the CV curves of different electrodes in the presence of 5.0 mM [Fe(CN)_6_]^3−/4−^ containing 0.1 M KCl. As it can be seen, compared to bare SPCE, the peak current decreased after coating the SPCE with streptavidin and further probe immobilization, which apparently blocked and reduced the electron transfer of [Fe(CN)_6_]^3−/4−^ to the electrodes. In contrast, after the addition of nanoprobe/miR-106a, the highest peak current was obtained, which was attributed to electro-conductivity properties of gold NPs that could accelerate the electron transfer [45].

### Optimization of the fabrication and biosensing procedure

In order to achieve optimal sensing performance, various important experimental parameters, including the incubation time, concentration and immobilization time for streptavidin and ss-probes, and also hybridization time were investigated.

In the case of preparation of gold-magnetic nanoprobe, various amounts of streptavidin (5–30 µg) and various coupling times (30–120 min) were separately examined. For 20 mg functionalized gold–magnetic NPs, an optimal amount of 20 µg streptavidin and a sufficient coupling time of 60 min were determined based on higher coupling efficacy (estimated by OD_280_ measurement, Figure S3A, Supporting Information File 1). Subsequently, by examining different amounts of P1 (100–500 pmol) and different immobilization time (30–120 min), optimal amount of P1 (400 pmol) and immobilization time (90 min) were also defined based on immobilization efficacy (Figure S3B, Supporting Information File 1).

Furthermore, the optimal assay parameters were explored based on the best DPV response in the presence of a miR-106a concentration of 10 pM with minimum reagent expenses. The immobilization of P2 on the SPCE surface also occurred through the streptavidin–biotin interaction. In this regard, different concentrations of P2 and immobilization times were assayed via DPV analysis. As the Figure S3C (Supporting Information File 1) shows, the results obtained for different amounts of the P2 within 120 min of immobilization time, revealed that the peak current increased with increasing amounts of the probe, reaching a current plateau for 0.3 ng/µL. Therefore, a saturation concentration of 0.3 ng/µL of the probe and the immobilization time of 60 min were chosen for subsequent studies.

A main consideration regarding biosensors based on the hybridization of nucleic acids is the hybridization time. The influence of the hybridization time was evaluated for optimum analytical performance. Various time periods (30–120 min) were examined for the hybridization steps between the target miRNA and each probe. Figure S3D (Supporting Information File 1) demonstrates that the DPV peak currents increased rapidly with the hybridization time up to about 45 min and then more slowly to reach a current plateau at about 60 min; thus, the incubation time for each hybridization step was chosen to be 60 min.

Condition time and potential used in the electrochemical assay also play important roles in biosensor performance. It is claimed that the electrode surface may be damaged at high potentials, whereas low potentials is too week to oxidize gold. Therefore, a constant potential of 1.3 V was selected according to literatures [20] and the DPV analysis was carried out with different peroxidation times (20–120 s). The experiments were then performed at different potentials (1–1.5 V). Based on the peak current intensity, 60 s and 1.20 V were finally chosen as the optimal preoxiditaion time and condition potential, respectively (Figure S3E and S3F, Supporting Information File 1).

### Catalytic performance of the miRNA-nanobiosensor

**Figure 4:**
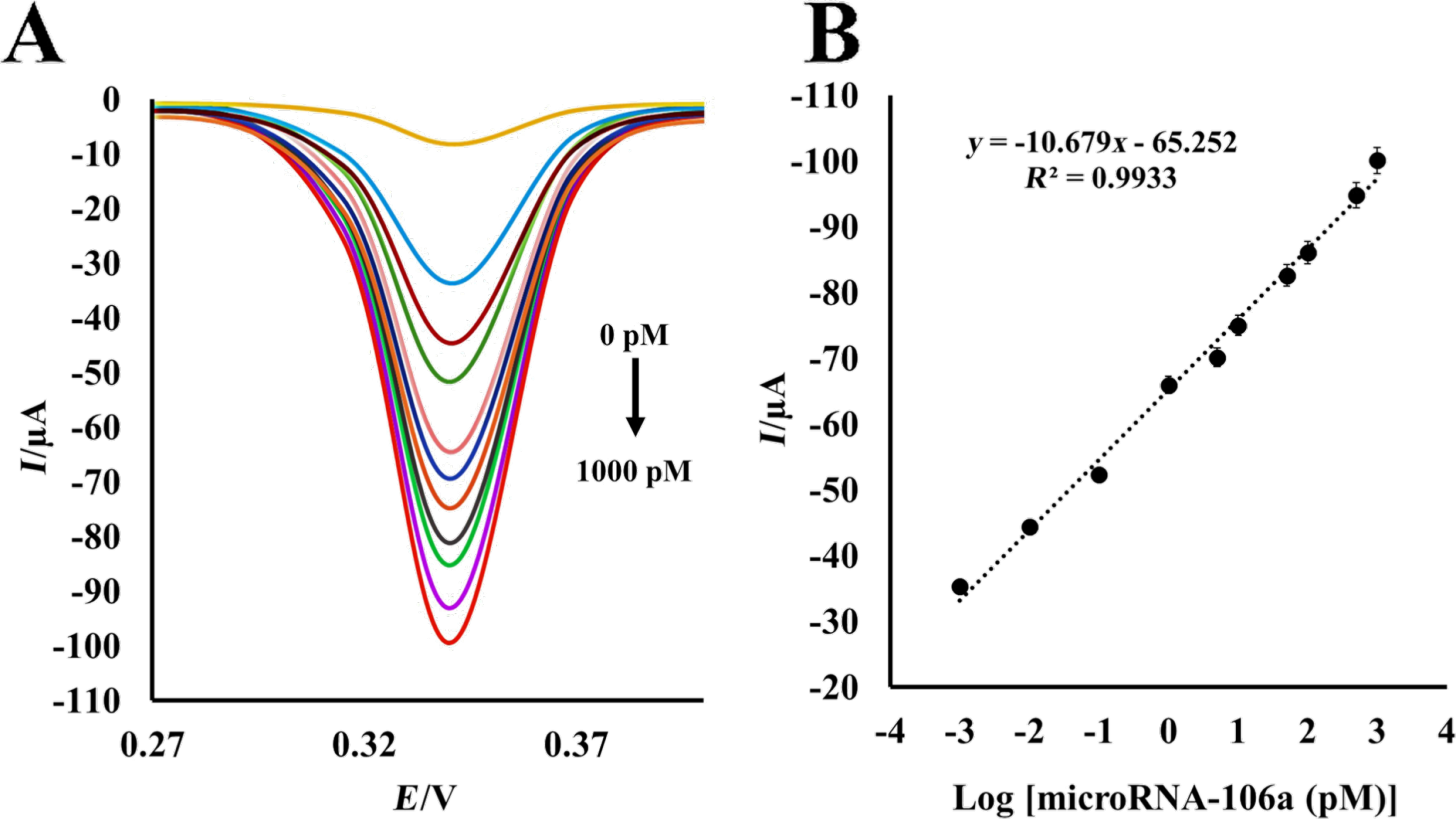
(A) Differential pulse voltammograms for the electrochemical detection of miR-106a upon serial dilutions of target miR-106a at scan rate 100 mV/s in 1 M HCl. The concentrations of target miRNA are: 0, 0.001, 0.01, 0.1, 1, 5, 10, 50, 100, 500, and 1000 pM. (B) The calibration curve of miR-106a as the relationship between current and logarithm of miR-106a concentration. Each data point is the average of five replicates.

The whole biosensing procedure was carried out at room temperature and physiological pH value. Under optimal conditions, different concentrations of miR-106a were assayed using the double probe hybridization-based methodology, in which gold-magnetic nanoprobe was employed as signal tag on the P2/streptavidin-modified SPCE as the sensing platform. Following optimization of the procedure, the DPV analysis was performed in five replicates for the evaluation of miR-106a in standard solutions prepared by diluting a known concentration of target miR-106a with healthy serum sample. DPV voltammograms were recorded between +0.1 V and +0.40 V at scan rate of 100 mV/s by holding the working electrode at a condition potential of +1.20 V for 60 s in HCl. As Figure 4A shows, the DPV peak currents noticeably enhanced with the increase of miR-106a concentrations in standard solutions. The logarithmic plot of the miR-106a concentration versus current was linear in the wide range from 1 × 10^−3^ pM to 1 × 10^3^ pM of the target miR-106a (Figure 4B) with the regression equation of *I* = −10.679[log *C*] − 65.252 (*I* is the peak current and *C* is the miRNA concentration, *R*^2^ = 0.9933). The detection limit of miR-106a nanobiosensor was also calculated to be 0.3 fM using the equation *C*_m_ = 3*sb*_1_/*m*, where *sb*_l_ is the standard deviation of 12 repeated DPV of the blank response and *m* is the slope of the calibration curve [46]. Furthermore, the mean reproducibility intra-assay CV (coefficient of variation) for 0.1 and 50 pM miR-106a were 3.2% and 2.2%, respectively, and the inter-assay reproducibility was 5.5% and 4.8%, respectively. These results demonstrate that the fabricated miRNA-nanobiosensor promises an efficient quantification process with trace detection limit and acceptable reproducibility, which is really valuable in biosensing analysis.

**Figure 5:**
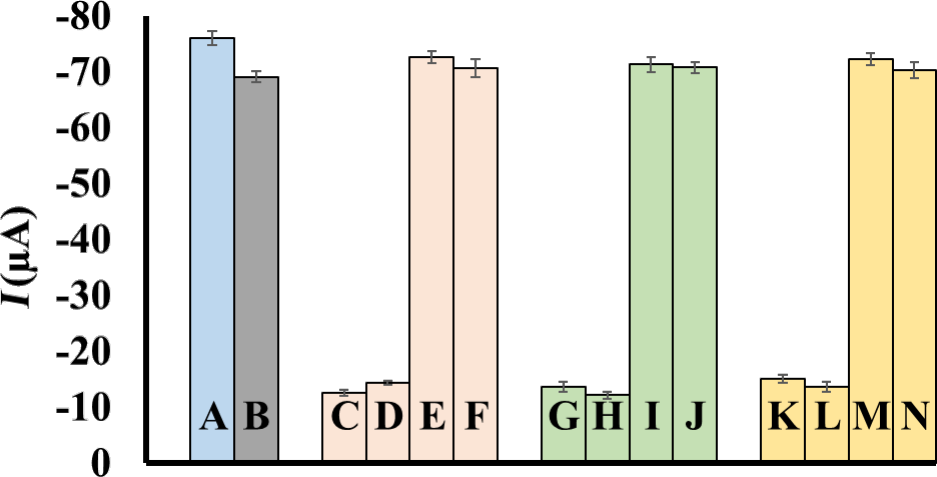
Differences in signal intensities in presence of interference miR-15a (nc1), miR-21 (nc2), and miR-200c (nc3). (A) miR-106a (10 pM); (B) miR-106a (10 pM) + nc1 (10 pM) + nc2 (10 pM) + nc3 (10 pM); (C) nc1 (10 pM); (D) nc1 (50 pM); (E) miR-106a (10 pM) + nc1 (10 pM); (F) miR-106a (10 pM) + nc1 (50 pM); (G) nc2 (10 pM); (H) nc2 (50 pM); (I) miR-106a (10 pM) + nc2 (10 pM); (J) miR-106a (10 pM) + nc2 (50 pM); (K) nc3 (10 pM); (L) nc3 (50 pM); (M) miR-106a (10 pM) + nc3 (10 pM); (N) miR-106a (10 pM) + nc3 (50 pM).

In addition, the selectivity of this nanobiosensor for miR-106a detection was evaluated and compared in presence and absence of three non-complementary miRNAs as interfering sequences. As Figure 5 shows, under the same conditions DPV responses generated from non-complementary miRNAs via non-specific binding, were considerably weaker. Moreover, it is evident that in presence of these non-complementary targets, even in a concentration about five times more than complementary target (miR-106a), minimal interference was detected in DPV peak currents. The DPV signals generated in presence of miR-106a demonstrated that the presented nanobiosensor is highly selective for miR-106a and is not considerably affected by the interfering sequences.

Moreover, the storage stability of modified electrodes was evaluated by comparing the electrochemical performance of the stored P2/streptavidin/SPCEs with the freshly-prepared ones over a 10-week period. As the results show (Figure S4), the response of miRNA-nanobiosensor remained quite stable up to eight weeks and after that, there was a loss in signal strength of about 30% [43,47].

### miR-106a quantification in spiked and real samples

The expression of cancer-associated miRNA-106a in serum can be screened as a valuable non-invasive biomarker of GC. However, quantification methodologies are only useful if they are able to detect miRNA in serum, despite of background noises generated from serum factors. Table 1 shows the analyses of spiked samples that were performed by the fabricated nanobiosensor with five different known concentrations of the target miR-106a added to human serum. The high recovery and low RSD values prove the obvious potential of this nanobiosensor for detecting miR-106a in serum environment.

**Table 1:** Spike and recovery results obtained from miRNA-nanobiosensor in serum. Data are presented as the means of five replicates.

sample number	miR-106a added (pM)	miR-106a found (pM)	RSD %	recovery %

1	1.0 × 10^−2^	1.03 × 10^−2^	4.13	103.0
2	1.0 × 10^−1^	1.04 × 10^−1^	3.40	104.0
3	1.0	9.86 × 10^−1^	4.09	98.60
4	10	10.1	2.41	101.0
5	1.0 × 10^2^	97.8	3.77	97.80
6	1.0 × 10^3^	9.89 × 10^2^	2.26	98.90

Furthermore, in order to confirm the applicability of our nanobiosensor for analyzing circulating miR-106a in real samples, human serum samples from ten newly diagnosed GC patients and ten healthy volunteers were tested. The recorded electrochemical signals presented in Table 2 demonstrate the upregulation of miR-106a in the serum samples of GC patients, which was anticipated according to the literature [2–3]. As references, the expression level of the serum miR-106a was simultaneously quantified by qRT-PCR after an extra RNA extraction step. Comparing the results obtained from both methods shows such a good and acceptable agreement.

**Table 2:** Comparison of miR-106a detection in serum samples of GC patients and healthy volunteers using the nanobiosensor and qRT-PCR. Data are presented as the means of five replicates.

cancerous serum samples	miR-106a nanobiosensor		qRT-PCR		healthy serum samples	miR-106a nanobiosensor		qRT-PCR	
	mean (pM)	RSD %	mean (pM)	RSD %		mean (pM)	RSD %	mean (pM)	RSD %

1	8.33	2.40	9.30	2.46	1	2.05	2.95	2.42	2.58
2	8.85	3.12	11.2	2.20	2	1.39	4.56	1.12	4.93
3	7.31	4.40	8.42	3.05	3	0.96	2.08	0.79	3.35
4	8.24	3.40	9.51	3.51	4	0.94	3.43	0.76	2.02
5	8.67	1.99	11.6	2.11	5	1.30	3.63	1.94	2.44
6	8.39	2.91	10.5	3.18	6	2.17	4.55	1.24	3.70
7	7.26	3.05	9.28	2.12	7	0.85	2.44	1.23	4.08
8	9.64	2.68	10.3	4.07	8	2.06	3.90	2.27	4.60
9	7.66	2.95	8.47	3.15	9	1.13	4.19	2.11	3.55
10	7.29	2.27	9.25	2.03	10	0.75	2.05	1.08	2.82
average	8.16		9.79		average	1.36		1.50	

It is worth noting that commonly used miRNA determination techniques such as northern blot and qRT-PCR provide a significantly low detection limit (aM-nM) and excellent selectivity [21]. However, their complex time-consuming procedure along with the need for amplification steps has made them inappropriate for routine clinical screening. In contrast, the results of presented nanobiosensor performance indicated the notable advantages of this device such as low-cost, simple preparation procedure, fast response, remarkable selectivity, high recovery, and good stability. Furthermore, in comparison with previously reported miRNA-(nano)biosensors, the presented nanobiosensor provides a simpler more time- and cost-effective methodology with a wide linear range and low limit of detection (Table 3). According to the remarkable performance in real biological environments (human serum sample) and no need for RNA extraction, pretreatment, or amplification, it is believed that the designed nanobiosensing platform can serve as an attractive candidate for direct and sensitive quantification of miRNA in clinical diagnosis especially with the aim of early cancer detection.

**Table 3:** The comparison of the proposed electrochemical nanobiosensor specifications with selected recently published electrochemical biosensors for the detection of miRNA.^a^

method	electrode/modification	target miRNA	brief biosensing strategy	linear range/detection limit	real sample analysis/related disease	ref

Amp	GE/ 3D tetrahedral scaffold probe	miR-122b	HCR-amplified hybridization signal and HRP-based signal generation	10 aM to 1 pM/10 aM	NA/NS	[48]
	GE/ CP	miR-21	combination of DSN with signal amplification of ALP and redox cycling reaction	NA/0.2 fM	NA/NS	[49]
	SPdCE	miR-21, miR-205	chitin-MB* baring p19 as CP/ HRP	2–10 nM/0.6 nM	total RNA extracted from cancer cell lines and tumor tissues/breast cancer	[50]
SWV	GE/PNA CP	let-7c	RuO_2_-initiated polymerization of aniline and miRNA-templated deposition of polyaniline	5 fM to 2 pM/2 fM	total RNA extracted from cultured cells /Lung cancer	[51]
EIS	GE/PNA CP	let-7b	hybridized miRNA-guided deposition of polyaniline	1 fM-5 pM/ 0.5 fM	RNA samples extracted from cancer cells and blood/NS	[52]
	GE/CP	let-7c	polymerization of DB in presence of DNAzyme and miRNA-templated deposition of PDB	5 fM to 1 pM/2 fM	total RNA extracted from cultured cells/lung cancer	[53]
	GE/hairpin probe	miR-26a	multiple-DNAzyme strategy/GNP tags/hemin	30 aM to 10 fM/15 aM	NA/NS	[33]
CV	GCE/nafion + thionine + Pd NPs + CP	miR-155	change of the amperometric response before and after the CP:target miRNA hybridization	5.6 pM to 56 µM/1.87 pM	spiked human serum samples/NS	[54]
	GE/CP	miR-499	intercalation of MB in miRNA:CP hybrid/HRP	1 pM to 100 nM/0.3 pM	spiked human serum samples/AMI	[55]
DPV	SPGE/stem-loop CP	miR-21	double axillary probes/RuHex	100 aM to 100 pM/100 aM	human serum samples/breast cancer	[56]
	GCE/ GO + GNR + CP	miR-155	intercalation of OB in miRNA:CP hybrid	2 fM to 8 pM/0.6 fM	spiked human plasma samples/breast cancer	[21]
	GCE/ MWCNT + CP	miR-21	intercalation of MB in miRNA:CP hybrid	0.1–500 pM/84.3 fM	NA/breast cancer	[30]
	GE/gold NP + ferrocene-tagged DNA of stem-loop structure combined with tetrahedron DNA nanostructure	miR-21	3D DNA origami structure	100 pM to 1 µM/10 pM	cell lysate/lung cancer	[57]
	SPCE/streptavidin + biotinylated CP	miR-106a	double-probe sandwich construction containing gold–magnetic NPs	1 fM to 1 nM/0.3 fM	human serum samples/gastric cancer	this work

^a^ALP: alkaline phosphatase, AMI: acute myocardial infarction, Amp: amperometry, CP: capture probe, CV: cyclic voltammetry, DB: 3,3′-dimethoxybenzidine, DPV: differential pulse voltammetry, DSN: duplex-specific nuclease, EIS: electrochemical impedance spectroscopy, GCE: glassy carbon electrode, GE: gold electrode, GNP: gold nanoparticle, GNR: gold nanorod, GO: graphene oxide, HCR: hybridization chain reaction, HRP: horseradish peroxidase, MB: methylene blue, MB*: magnetic bead, MWCNT: multiwalled carbon nanotube, OB: oracet blue, NA: not analyzed, NP: nanoparticle, NS: not specified, PDB: poly(3,3′-dimethoxybenzidine), PNA: peptide nucleic acid, RuHex: hexaamineruthenium(III) chloride, SPCE: screen-printed carbon electrode, SPGE: screen-printed gold electrode, SPdCE: dual screen-printed carbon electrode, SWV: squarewave voltammetry.

## Conclusion

In this study, we have reported the construction of a miRNA-nanobiosensor that can quantify the miR-106a expression level by benefiting from the electrochemical properties of a gold–magnetic nanoprobe as signaling label. The proposed nanobiosensor accurately monitors miRNA targets in real serum samples. Simple handling, short assay time, good reproducibility, low detection limit, wide linear range, considerable selectivity, and especially no need for sample pretreatment, present this miRNA biosensing procedure as an ideal way to detect and evaluate miR-106a in biological samples for clinical application. It is believed that this miRNA-nanobiosensor could be used for early detection of GC and also for assessing the efficiency of therapies.

## Experimental

### Reagents and chemicals

Synthetic ss-DNA probes and miRNA sequences employed in this study were purchased from Bioneer Corporation (South Korea) and the sequences are listed in Table S1. Additionally, ferric chloride hexahydrate (FeCl_3_·6H_2_O), ferrous chloride tetrahydrate (FeCl_2_·4H_2_O), and acetic acid were obtained from Acros Organics (USA). Chloroauric acid (HAuCl_4_), BSA, sodium dodecyl sulfate (SDS), low molecular weight chitosan, dialysis tube with molecular cut off 12000 Da, sodium azide, KCl, 1-ethyl-3-(3-dimethylaminopropyl)carbodiimide (EDC), *N*-hydroxysuccinimide (NHS), 11-mercaptoundecanoic acid (MUA), streptavidin, K_3_Fe(CN)_6_, and K_4_Fe(CN)_6_ were purchased from Sigma Chemical Company (USA). Analytical grade HCl, NaCl, D-glucose, glutaraldehyde, *N*-methylpyrrolidone (NMP), iodomethane, sodium hydroxide, sodium iodide and acetone were purchased from Merck (Darmstadt, Germany). All other chemicals employed were of analytical grade and deionized water was utilized in all the experiments.

Buffers used in the experiment included Tris buffer (including 2% BSA), the immobilization buffer (NaCl 300 mM, Na_2_HPO_4_ 20 mM, EDTA 0.1 mM at pH 7.4), and the hybridization buffer (NaCl 300 mM, sodium citrate 30 mM at pH 7.4).

The SPCEs (3.4 cm length × 1.0 cm width × 0.05 cm height) were purchased from DropSens (Spain). The SPCE consists of a 4 mm diameter disk carbon working electrode, carbon counter electrode, and a silver pseudo-reference electrode.

### Synthesis and characterization of gold–magnetic NPs

Gold–magnetic NPs were synthesized as a three-layer nanostructure (gold@TMC@Fe_3_O_4_) according to the protocol described elsewhere [36]. In short, all the components (gold and magnetic NPs, and TMC polymer) were separately prepared. In order to synthesize the Fe_3_O_4_ NPs, Fe(II) and Fe(III) chloride (Fe(II)/Fe(III) ratio of 0.5) were coprecipitated in the presence of NaOH. The *N*-trimethylchitosan (TMC) polymer was produced using iodomethane, *N*-methylpyrrolidone and NaOH according to the literature. Colloidal gold NPs were synthesized using D-glucose as the reducing agent via a typical short procedure at 60 °C [36].

In the following, 200 mg of magnetic NPs was exposed to appropriate amount of previously produced TMC (Fe_3_O_4_/TMC molar ratio of 1) in a 20 mL solution containing glutaraldehyde (25%, 2 mL), NaCl (0.5 M), and SDS (0.025 M). The TMC-coated magnetic NPs were produced through stirring at 50 °C for 5 h. Next, these TMC@Fe_3_O_4_ NPs were mixed with 25 mL of freshly prepared gold NPs; and the gold–magnetic nanocomplexes were finally obtained after stirring for 1 h at room temperature [36].

The final NPs were then subjected to standard characterization analyses to evaluate whether they meet the essential criteria. For this purpose, the size and morphology of particles were estimated by means of transmission electron microscopy (TEM) (Zeiss, EM10C, 80 kV, Germany) and dynamic light scattering (DLS) analysis (Zetasizer nano-ZS, Malvern, England). The NPs were also analyzed using UV–vis spectroscopy (Lambda 950 UV–vis spectrophotometer, Perkin Elmer, USA) at about 520 nm after each preparation step. The magnetic behavior of gold–magnetic NPs was investigated via vibrating sample magnetometer (VSM) (MAG-3110, Freescale) analysis. In order to study structural and elemental properties, X-ray diffraction (XRD) (Philips, pw 1800, Netherlands) and energy-dispersive X-ray diffraction (EDX) were carried out. These NPs were also characterized using Fourier transform infrared spectroscopy (FTIR) to study the chemical interactions in the product formulation especially during covering the magnetic core with TMC polymer.

### Preparation of gold–magnetic nanoprobes

The biotin-labeled probe P1 was attached onto the gold–magnetic NPs via a three step process: (1) functionalization of gold–magnetic NPs; (2) coating the functionalized gold–magnetic NPs with streptavidin; and (3) P1 immobilization onto the NPs.

First, in order to carboxylate the gold–magnetic NPs, 100 mg of these particles were added to 1 mL ethanol solution of 20 mM MUA and incubated overnight after 1 h of sonication. Second, streptavidin was covalently immobilized on the surface of these NPs by linking the streptavidin amine groups to the carboxylic acid groups on the functionalized particles. A 20 mg portion of gold–magnetic NPs bearing carboxylic groups was dispersed into the 1 mL solution of EDC (10 mM) and NHS (20 mM) prior to sonication for 20 min at 4 °C. Afterward, the particles were exposed to 20 µg of streptavidin and the volume was adjusted to 500 µL with phosphate buffer saline (PBS) (0.01%, pH 7.4). The resulting suspension was incubated under shaking condition at room temperature for 1 h. The particles were washed and magnetically separated. In order to evaluate the coupling efficiency, OD_280_ values of the streptavidin solution of pre-coupling and post-coupling were determined by an ultraviolet–visible spectrometer. In the last step, in order to immobilize biotinylated P1 on the streptavidin-coated NPs, these NPs were incubated with 1 mL immobilization buffer containing 400 pmol of P1 at 37 °C for 1.5 h. Finally, the particles were washed with immobilization buffer and OD_260_ values of the oligonucleotide solution for pre- and post-coupling were measured to calculate immobilization efficiency [42].

### Electrode modification

In order to physically absorb streptavidin onto the electrode surface, a 10 µL portion of PBS containing 2 µg streptavidin was dropped on the working zone of each SPCE. After overnight incubation at 4 °C, the streptavidin-modified SPCEs were washed with Tris buffer to remove the excess protein. Free surface sites were then blocked using BSA (2%, 40 µL) for 15 min. After washing the SPCEs by Tris buffer, the electrodes were subjected to immobilizing P2 on their surfaces. For this purpose, 40 µL of biotin-labeled P2 (0.30 ng/µL) was added to the working space of SPCEs and the electrodes were incubated for 60 min at room temperature [58]. The SPCEs were also subjected to atomic force microscopy (AFM) analysis for studying the morphology changes during modification process.

### Quantification of miR-106a by miRNA-nanobiosensor

#### Hybridization

The two hybridization steps were performed at room temperature. During the first step, 2 mL of hybridization buffer containing miRNA-106a was exposed to 3 mg gold–magnetic nanoprobes and incubated for 1 h under gentle shaking. The NPs were then washed using an external magnet. Next, the nanoprobe/miR-106a hybrid was resuspended in 30 µL of hybridization buffer and placed on the modified SPCEs to perform the second hybridization step. After 1 h of incubation, the electrodes were washed with Tris buffer and investigated through electrochemical measurements [42,58].

#### Electrochemical measurement

Cyclic voltammetry (CV), differential pulse voltammetry (DPV) and impedimetry methods were used to characterize the stepwise fabrication of the miRNA-nanobiosensor. The basic analysis of the SPCEs in this study was performed via CV mode, at the potential range of −0.7 to +1.1 V and the scan rate of 50 mV/s using 5.0 mM [Fe(CN)_6_]^3−/4−^ containing 0.1 M KCl. The performance of the biosensor was evaluated by DPV mode. The electrochemical detection involved oxidation of the gold NPs at the constant potential of +1.20 V for 60 s and subsequent reduction to Au^0^ while scanning from +0.4 V to +0.1 V with a step potential of 4 mV, a pulse amplitude of 50 mV and a pulse time of 20 ms [40].

In addition, the performance of the nanobiosensor in detecting miR-106a was assessed in the presence and the absence of miR-106a and two concentrations of non-complementary sequences using DPV. After preparing the sample solutions, the biosensing procedure was carried out and the DPV peak currents were recorded according to the parameters mentioned above.

Furthermore, the stability of modified electrodes was investigated after 10 weeks storage in 4 °C and compared to that of freshly prepared electrodes. The reproducibility of the miRNA-nanobiosensor performance was also evaluated through quantification of two concentrations in eight independent assays.

#### Quantification of miR-106a by stem-loop RT-PCR

After preparing a serial dilution of miR-106a, cDNA was synthesized using s stem-loop primer and M-MuLV reverse transcriptase, and then quantified on an Applied Biosystems 7900HT sequence detection system by using a SYBR® Premix Ex Taq™ II (TAKARA BIO INC.) according to the protocol of the manufacturer. The obtained Ct values were considered for plotting the standard curve.

#### Spiked sample analysis

In order to study the nanobiosensor performance in biological fluids, the serum sample was used instead of hybridization buffer. Therefore, different defined concentrations were prepared by adding synthesized miR-106a into the healthy non-cancerous serum sample. Using the proposed miRNA-nanobiosensor, these samples were analyzed in five replicates following the previously explained biosensing methodology. The recovery of experiment was calculated as well as the relative standard deviation (RSD) values for each concentration.

#### Detection of miR-106a in serum samples of GC patients

The whole blood samples were primarily collected from 10 healthy persons and 10 patients with GC. For isolating the serum, the blood samples were allowed to clot in the absence of anticoagulant and simply centrifuged with recovery of supernatant. The serum samples were kept as 300 µL aliquots at −80 °C until further processing [59]. Using a Trizol-based method, total miRNA was isolated and subjected to qRT-PCR (as the gold standard method) for miR-106a detection. The same volume of serum samples was simultaneously analyzed via miRNA-nanobiosensor with no pretreatment, amplification, or RNA isolation.

## Supporting Information

File 1 Additional experimental data.
