# Supplementary material for: A novel electrochemical nanobiosensor for the ultrasensitive and specific detection of femtomolar-level gastric cancer biomarker miRNA-106a
Source: Beilstein J Nanotechnol. 2016 Dec 19;7:2023–36. doi: 10.3762/bjnano.7.193 (PMC5238648; doi:10.3762/bjnano.7.193)
Supplement: File 1 — Additional experimental data. [file Beilstein_J_Nanotechnol-07-2023-s001.pdf]

Supporting Information

for

A novel electrochemical nanobiosensor for the ultrasensitive and specific detection of femtomolar-level gastric cancer biomarker miRNA-106a

Maryam Daneshpour[1], Kobra Omidfar*[2,3,§] and Hossein Ghanbarian*[,4,5,¶]

Address: [1]Biotechnology Department, School of Medicine, Shahid Beheshti University of Medical Sciences, Tehran, Iran, [2] Biosensor Research Center, Endocrinology and Metabolism Molecular-Cellular Sciences Institute, Tehran University of Medical Sciences, Tehran, Iran, [3]Endocrinology and Metabolism Research Center, Endocrinology and Metabolism Research Institute, Tehran University of Medical Sciences, Tehran, Iran, [4]Cellular and Molecular Biology Research Center, Shahid Beheshti University of Medical Sciences, Tehran, Iran, and [5]Department of Biotechnology, School of Advanced Technologies in Medicine, Shahid Beheshti University of Medical Sciences, Tehran, Iran

Email: Kobra Omidfar* - omidfar@tums.ac.ir; Hossein Ghanbarian* - hghanbarian@sbmu.ac.ir

* Corresponding author

[§]P.O. Box: 14395/1179; Tel: +98 21 8822003738, Fax: +98 21 88220052

[¶]P.O. Box: 19395/4719; Tel: +98 21 22439957, Fax: +98 21 22439956

Additional experimental data

Table S1: The sequences of the used oligonucleotides in this study.

**Probe 1 (P1)**	5'-TGTAAGCACTTTT-3'-$(CH_2)_6$-biotin
**Probe 2 (P2)**	biotin-$(CH_2)_6$-5'-CTACCTGCAC-3'
**Target miR-106a**	5'-AAAAGUGCUUACAGUGCAGGUAG-3'
**Non-complementary target (miR-15a, nc1)**	5'-UAGCAGCACAUAAUGGUUUGUG-3'
**Non-complementary target (miR-21, nc2)**	5'-UAGCUUAUCAGACUGAUGUUGA-3'
**Non-complementary target (miR-200c, nc3)**	5'-CGUCUUACCCAGCAGUGUUUGG-3'

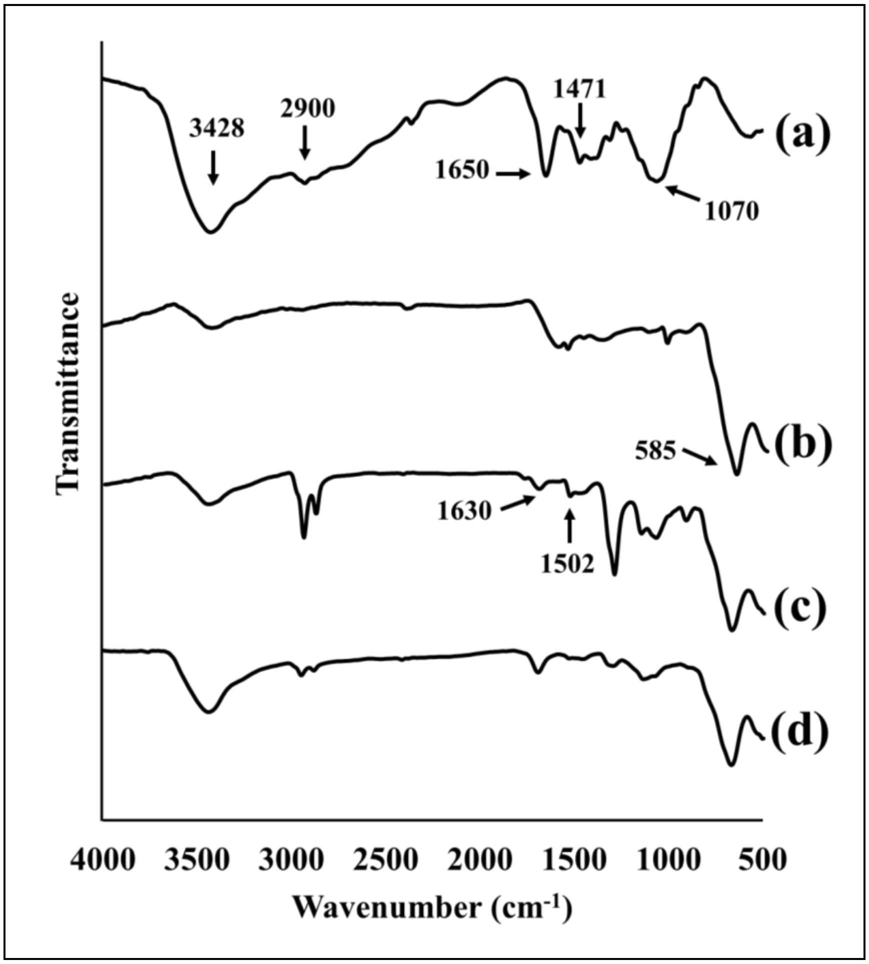

Figure S1: FTIR spectra of TMC polymer (a), Fe_3O_4 NPs (b), TMC@Fe_3O_4 NPs (c), and gold@TMC@Fe_3O_4 NPs (d).

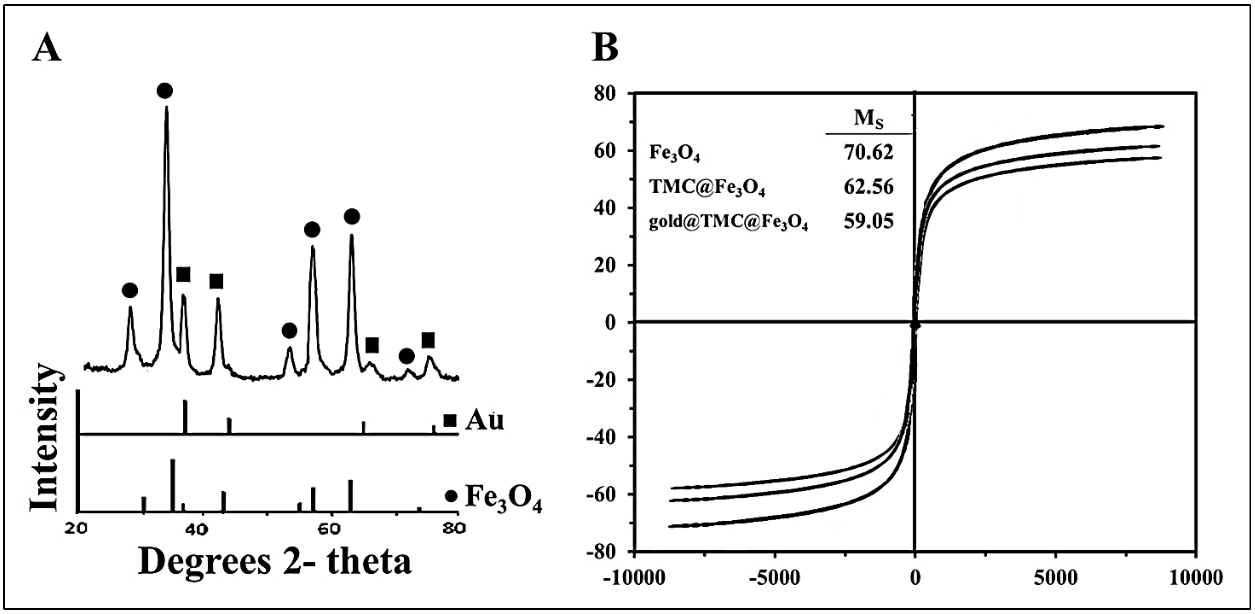

Figure S2: (A) X-ray diffraction pattern of gold-magnetic NPs. (B) Hysteresis loop of the synthesized NPs.

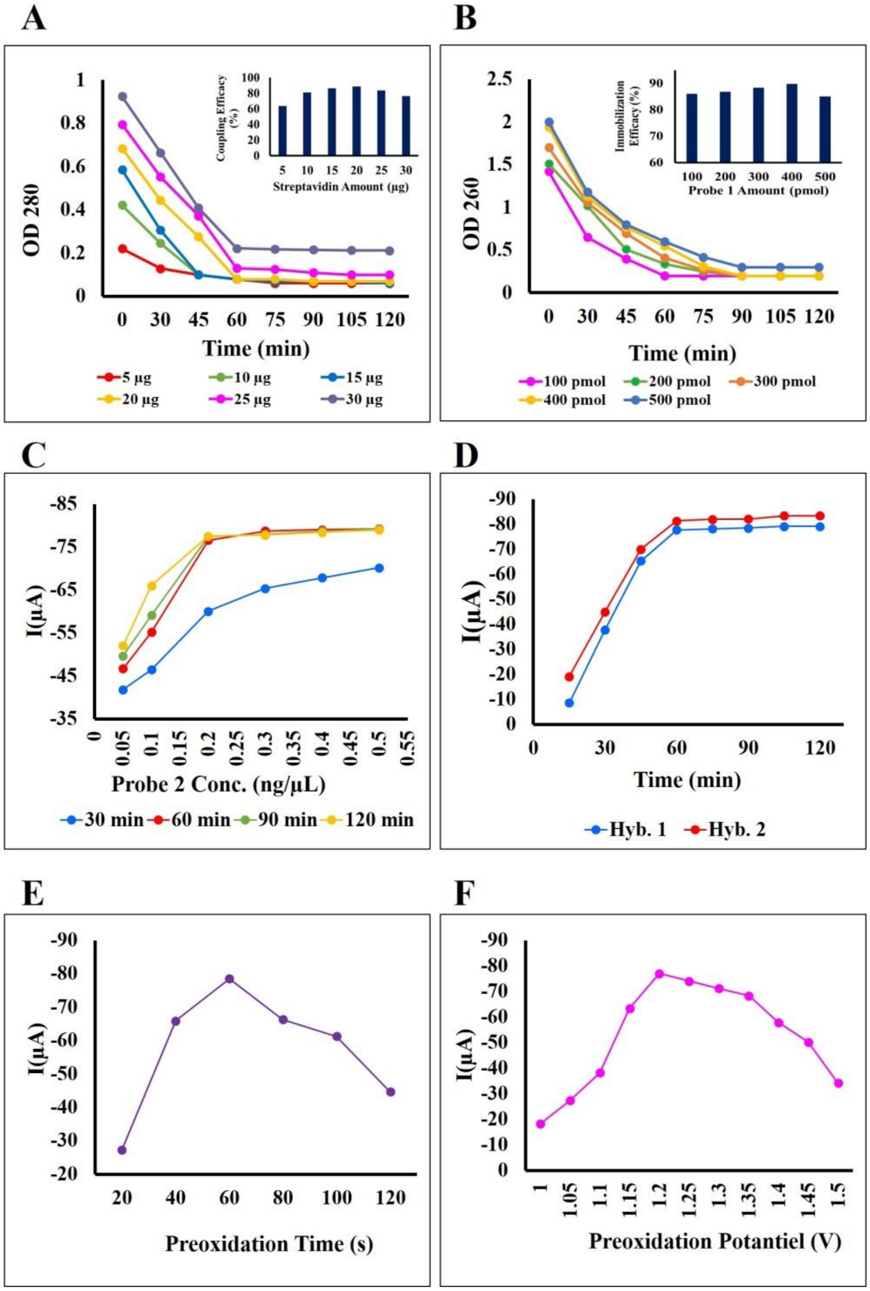

Figure S3: Optimization of the fabrication and biosensing procedure. Concentration and incubation time of (A) Streptavidin, (B) P1, and (C) P2. (D) The time of hybridization steps. Effects of (E) preoxidation time, and (F) potential between 1.0 and 1.5 V on DPV response of miRNA-nanobiosensor.

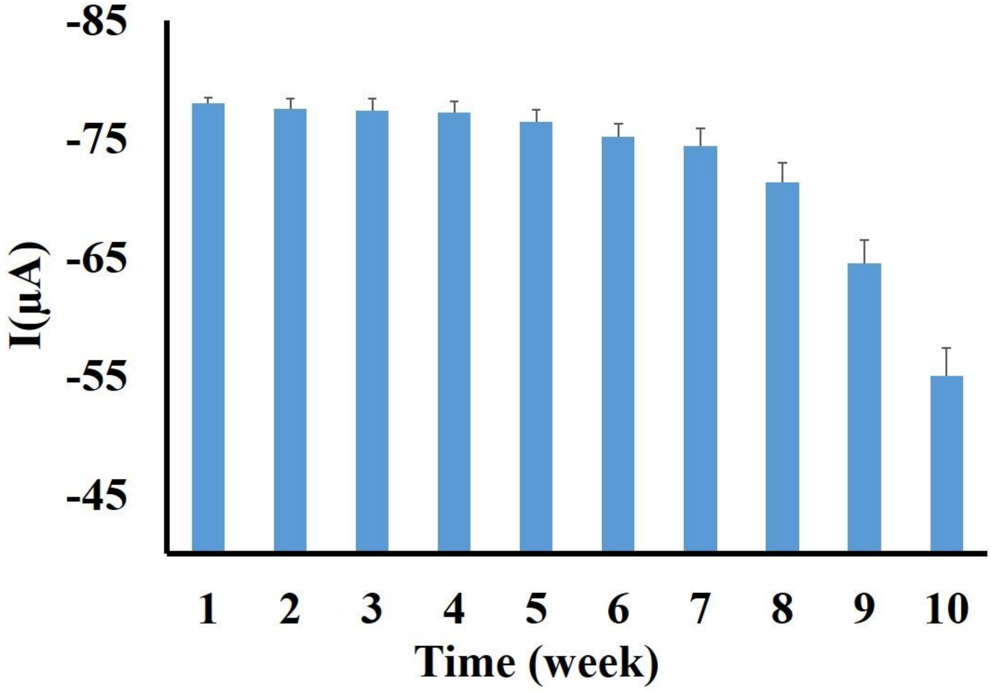

Figure S4: The stability of the modified electrodes after 10 weeks storage at 4 °C.
